# MA‐cont:pre/post effect size: An interactive tool for the meta‐analysis of continuous outcomes using R Shiny

**DOI:** 10.1002/jrsm.1592

**Published:** 2022-08-01

**Authors:** Katerina Papadimitropoulou, Richard D. Riley, Olaf M. Dekkers, Theo Stijnen, Saskia le Cessie

**Affiliations:** ^1^ Clinical Epidemiology Leiden University Medical Center Leiden The Netherlands; ^2^ Centre for Prognosis Research Research Institute for Primary Care & Health Sciences, Keele University Keele UK; ^3^ Biomedical Data Sciences Leiden University Medical Center Leiden The Netherlands

**Keywords:** ANCOVA, baseline imbalance, pseudo individual participant data, shiny

## Abstract

Meta‐analysis is a widely used methodology to combine evidence from different sources examining a common research phenomenon, to obtain a quantitative summary of the studied phenomenon. In the medical field, multiple studies investigate the effectiveness of new treatments and meta‐analysis is largely performed to generate the summary (average) treatment effect. In the meta‐analysis of aggregate continuous outcomes measured in a pretest‐posttest design using differences in means as the effect measure, a plethora of methods exist: analysis of final (follow‐up) scores, analysis of change scores and analysis of covariance. Specialised and general‐purpose statistical software is used to apply the various methods, yet, often the choice among them depends on data availability and statistical affinity. We present a new web‐based tool, MA‐cont:pre/post effect size, to conduct meta‐analysis of continuous data assessed pre‐ and post‐treatment using the aforementioned approaches on aggregate data and a more flexible approach of generating and analysing pseudo individual participant data. The interactive web environment, available by R Shiny, is used to create this free‐to‐use statistical tool, requiring no programming skills by the users. A basic statistical understanding of the methods running in the background is a prerequisite and we encourage the users to seek advice from technical experts when necessary.

## 1 INTRODUCTION

Meta‐analysis is a widely adopted methodology to synthesise findings from multiple studies investigating the same research topic, to provide a numerical summary of the studied topic and a measure of its uncertainty.^1^, ^2^, ^3^ In the medical setting, various research groups plan and conduct individual studies, often examining treatment effectiveness of new interventions (most commonly compared to a control/no treatment). There is, thus, vast amount of clinical evidence for healthcare practitioners and policy makers to keep pace with and often the evidence may additionally be contradictory.^4^ The need to accumulate the ever‐increasing medical evidence has led to the development of the meta‐analysis framework and its numerous methodological advances over the years.^5^

The widespread adoption of meta‐analysis by many research fields has increased the need for robust statistical tools for analysis. In the meta‐analysis of continuous outcome data (measured on the same scale), a plethora of analytic methods exist, which can be implemented in specialised and generic statistical software. Often choosing an analytic method boils down to the researcher's statistical and programming skills on a software of choice, and data availability.

In this work, we present a web‐based interactive tool — MA‐cont:pre/post effect size — we developed for meta‐analysing continuous outcomes to enable researchers perform appropriate analyses and present their results in a straightforward and meaningful fashion. The tool is open‐source, easily accessible from any internet browser (https://katerina-pap.shinyapps.io/MA-cont-prepostES/), thanks to R,^6^ RStudio^7^ and Shiny (an R package for building interactive web apps).^8^ The remainder of the paper is organised as follows: we briefly discuss the need for this tool and why it is important to have a user‐friendly software that allows for data pre‐processing (via algebraic calculations of imputations) followed by appropriate modelling approaches. Then, we introduce the tool, its objectives and functionalities based on R packages for data analysis and Shiny. We additionally provide an illustration of the tool, using a worked example and a step‐by‐step navigation through the functionalities of the application (app). We conclude with a discussion on future work.

## 2 WHY IS THERE A NEED FOR THIS TOOL?

The meta‐analysis of continuous outcomes measured at baseline and follow‐up can be performed by pooling the mean differences based on (a) follow‐up scores, (b) change scores, calculated by subtracting the follow‐up from the baseline score and (c) an analysis of covariance (ANCOVA) model, which adjusts for the baseline scores. Currently, the bulk of meta‐analysis of such continuous outcomes synthesise mean difference estimates obtained by the former two approaches, because ANCOVA estimates are rarely reported.^9^ However, when baseline imbalance occurs the conclusion of the meta‐analysis may shift depending on the analytic method.^10^

As long as the trials included in the meta‐analysis are *adequately* randomised, that is, it is not expected to be any systematic difference at baseline between the active and control groups, all three methods, based on follow‐up scores, change scores and ANCOVA will give similar unbiased estimates of the mean difference, yet the ANCOVA estimator is preferred for being more efficient.^11^, ^12^, ^13^, ^14^ However, even in randomised studies, chance baseline imbalance will always occur, which can only be taken into account by the ANCOVA; both follow‐up and change scores methods fall short as the former entirely ignores the baseline scores and the latter ignores the correlation between baseline and final scores. In addition, change scores are (negatively) correlated with baseline scores, which may produce an inflated treatment effect when more severe participants at baseline were assigned to the active treatment group.^15^ Thus, in the absence of reported ANCOVA estimates, the meta‐analyst is left between a choice of two estimates that cannot handle any (chance) baseline imbalance. Depending on the strength of the within‐group correlation between baseline and follow‐up scores, the mean difference estimates based on follow‐up and change scores will be closer to/farther from the ANCOVA estimate.

We have recently discussed options to recover ANCOVA estimates via available summary statistics under certain assumptions, for example, equal baseline/follow‐up scores correlations between the two groups (Papadimitropoulou under review,^11^, ^16^). In addition, we have proposed a novel approach, based on *sufficient statistics*, to perform ANCOVA meta‐analysis by generating and analysing pseudo individual participant data (IPD).^17^ This pseudo IPD approach provides identical results to the original IPD, as long as the pseudo IPD set matches the appropriate summary data for an ANCOVA.

While ANCOVA approaches have been recommended,^12^, ^18^ we believe that the lack of statistical expertise or software skills may be a barrier for non‐technical experts to catch‐up with these ANCOVA methods. A strong motivation behind the development of MA‐cont:pre/post effect size was thus, to enable a large audience of technical and non‐technical researchers to use the appropriate methods for their meta‐analysis. A larger aspiration and the reason why we provide the results of follow‐up scores, change scores approaches and various modelling options under the pseudo IPD approach, is to engage the user in a thinking journey to explore the different methods (and results) based on the assumptions on their data.

## 3 THE MA‐CONT:PRE/POST EFFECT SIZE TOOL

The app can be accessed via the following url: https://katerina-pap.shinyapps.io/MA-cont-prepostES/. All code is available in a github repository: https://github.com/Katerina-Pap/MA-cont-shiny-app.

**FIGURE 1:**
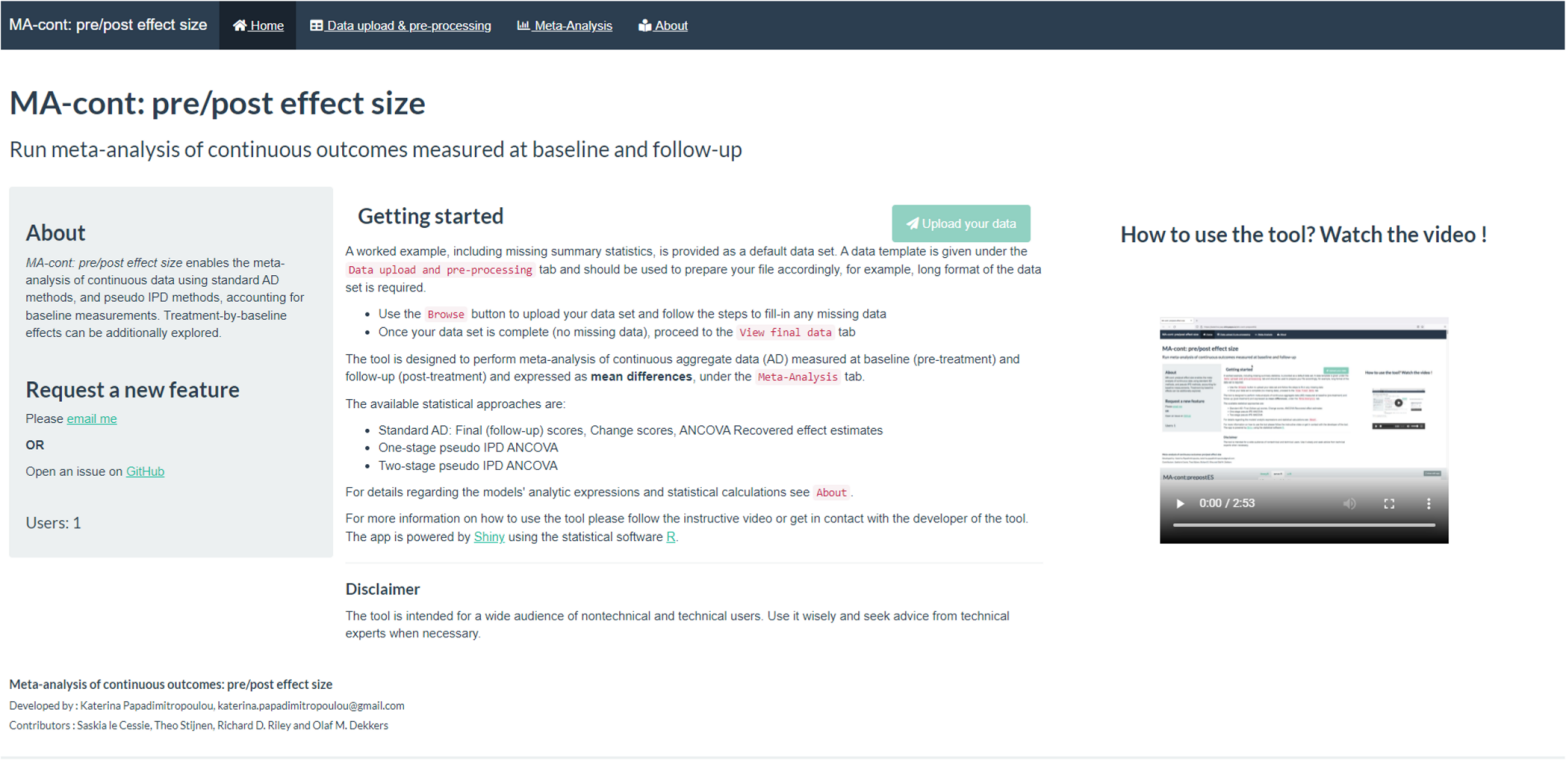
Homepage [Colour figure can be viewed at wileyonlinelibrary.com]

The tool is designed to make routinely used and more sophisticated meta‐analytic approaches available to the user, requiring no programming skills, but a good understanding of meta‐analytic concepts. The app offers a worked example, where a default data set is used to showcase the various functionalities. The users can upload their data sets into the webpage and proceed with data manipulation and analysis steps. All generated tabular and graphical output may be downloaded and accessed at a later time by the user. A step‐by‐step demonstration of MA‐cont:pre/post effect size is provided in a video of instructions found in the homepage of the tool (Figure 1).

### 3.1 Development

We used computing technologies and R packages, that is, RStudio,^7^ Shiny ^8^ and the extensively used packages for fitting

1. meta‐analytic models — metafor, ^19^
2. (non‐linear) mixed models — nlme. ^20^

A Shiny app executes R code on the backend; in this app most analyses are powered by metafor and nlme, without any requirements of local installation of R/RStudio. What the user sees in the browser is the frontend, the interface for the user to interact with the app via a personal computer, tablet or phone.

We encourage the reader/user to submit feedback on the existing functionalities and to provide suggestions for further improvement at https://github.com/Katerina-Pap/MA-cont-shiny-app/issues. We also welcome incremental updates by interested users and developers.

### 3.2 Statistical analysis

The tool offers five approaches to estimate the summary mean difference: the standard aggregate data (AD) methods of pooling follow‐up scores and change scores estimates, the ANCOVA recovered effect estimates and the one‐stage and two‐stage pseudo IPD ANCOVA. More details on the analytic expressions of the standard AD approaches and the ANCOVA recovered estimates approach can be found in Mckenzie et al.,^16^ Riley et al.^21^ and Papadimitropoulou (under review).

For each of the AD analytic methods, a choice between a random‐effects (RE) or a common (fixed)‐effect (CE) model^1^, ^2^ is provided to the user (default option is the RE). When RE models are fitted on the AD, or in the second step of the two‐stage pseudo IPD approach, the between‐study heterogeneity parameter *τ* ^2^, is estimated using the restricted maximum likelihood approach (REML). Simulation studies suggest that this estimator has recommendable properties over other iterative and non‐iterative estimators.^22^, ^23^, ^24^ In addition, under the RE model it is possible to obtain the refined standard error estimate of the summary treatment effect proposed by Hartung et al.^25^ and Sidik et al.^26^

Details on model formulation of the one‐ and two‐stage pseudo IPD ANCOVA approaches can be found in Papadimitropoulou et al,^17^ Papadimitropoulou (under review). In principle, one‐stage (pseudo) IPD meta‐analysis offers a plethora of modelling options and choices for a researcher educated in linear mixed models. A typical statistical dilemma is to choose between study‐stratified or random study intercepts to allow for the within‐trial clustering. Legha et al.^27^ have provided an excellent simulation study showing that adopting either approach results in minor differences in the summary estimates. In this tool, we offer only the option of study‐stratified intercepts, mainly because the mean difference estimate under this model naturally compares to the estimate obtained by the two‐stage pseudo IPD ANCOVA approach, where an ANCOVA is fitted per trial in the first step, and in the second step the derived treatment group coefficients (and their respective standard errors) are pooled. We allow more flexibility concerning the within‐trial residual variances, estimated under the one‐stage pseudo IPD approach. We provide four modelling options for the residual variances, possibly allowing more realistic scenarios, for example, residual variances varying by group but not by study (group‐specific).^17^, ^28^, ^29^

MA‐cont:pre/post effect size additionally enables the estimation of the within‐trial treatment‐by‐baseline interaction effect, which is rather straightforward when (pseudo) IPD are available. Appropriate adjustments to the one‐stage model are made to separate out within‐trial and between‐trial effects.^30^, ^31^

## 4 DEMONSTRATION OF MA‐CONT:PRE/POST EFFECT SIZE TOOL

We use an example data set of a meta‐analysis investigating the effect of calcium supplementation on reducing body weight,^32^ which also serves as the default data set in the app. The data set is comprised of the reported AD of nine randomised controlled trials comparing calcium supplements to placebo/no treatment and body weight measurements were taken at baseline and follow‐up time points. In addition, baseline imbalance in favour of the active treatment is present in these data.

As discussed earlier, the appropriate approach to synthesise such data is the ANCOVA, and thus per trial and per group, the means and standard deviations of baseline and follow‐up measurements, and the within‐group correlation should be ideally reported and/or obtained by other summary statistics.^33^

Step 1: Input data.

**FIGURE 2:**
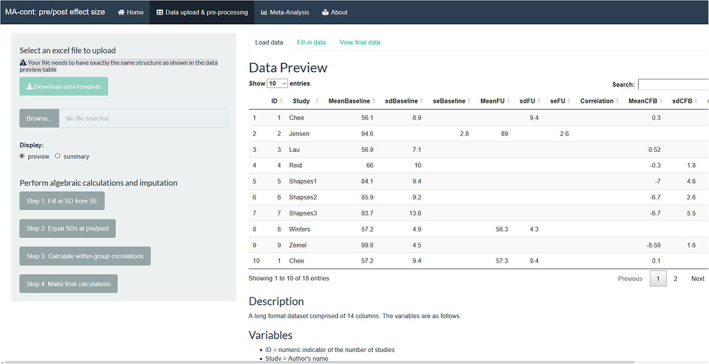
Screenshot of the data entry step for the default data set in long format and the output of data table [Colour figure can be viewed at wileyonlinelibrary.com]

The ‘Load data’ navigation tab allows the importing of the data set, in a long format and saved as an excel (.xlsx, .xls) file. The structure of the data set is specific, where the order of the variables has to match the one from the default data set (Figure 2). The users are encouraged to download the template file and prepare their data accordingly while preserving the order and the headers of the variables.

The output of this step is: (a) a data table showing the input data (in this case there are missing data therefore some table cells are blank) (b) a descriptive summary output, outlining missingness rate and key descriptive statistics (mean, standard deviation, median, etc) for each column. The user can obtain either output by clicking at the respective choice under the ‘Display’ button. A brief description of the variables is given under both options.

Step 2: Fill‐in any missing summary data.

**FIGURE 3:**
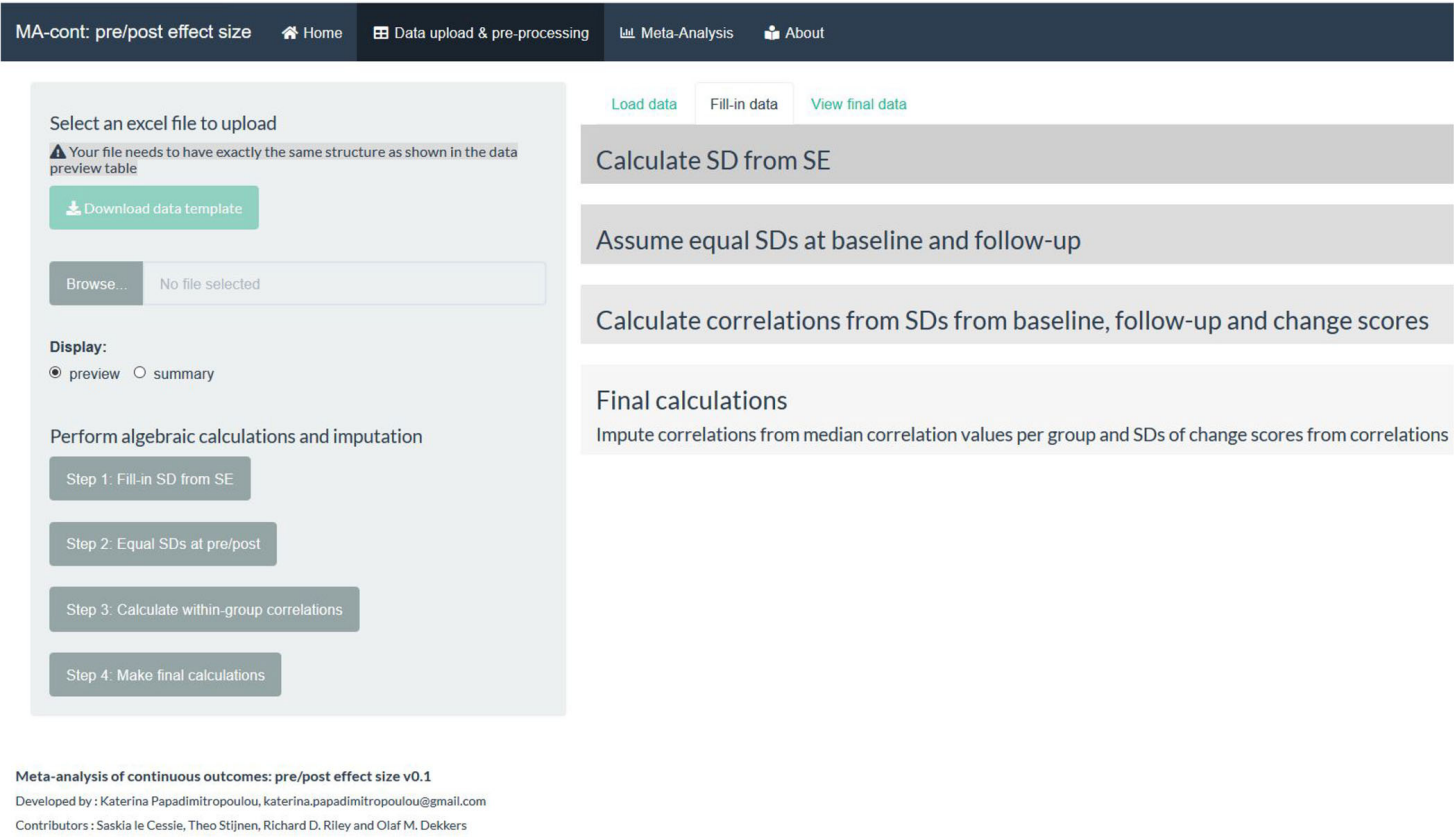
Screenshot of the ‘Fill‐in’ tab that enables stepwise calculation/imputation of missing summary data [Colour figure can be viewed at wileyonlinelibrary.com]

Often in the meta‐analysis of pretest‐posttest design trials, missing summary data exist; most commonly missing standard deviations and/or within‐group correlations. The tool offers a sequence of steps involving calculations and sensible imputations to fill‐in any missing data by clicking in the respective *action buttons* (Figure 3). For example, the *action button* ‘Fill‐in SD from SE’ calculates any missing standard deviations at baseline, follow‐up, of change scores as the product of the respective known standard errors and the square root of the sample sizes. Similarly, by clicking on the rest of *action buttons* (in the order they are presented), a full data set is constructed by first assuming any missing standard deviations at follow‐up equal to baseline standard deviations and thus the calculation of the within‐group correlation is possible by the following formula^33^:

$$r=\frac{{\mathrm{sd}}_{B}^{2}+{\mathrm{sd}}_{F}^{2}-{\mathrm{sd}}_{\text{Changescores}}^{2}}{2{\mathrm{sd}}_{B}{\mathrm{sd}}_{F}},$$

where suffixes *B*, *F* stand for baseline and follow‐up, respectively.

**FIGURE 4:**
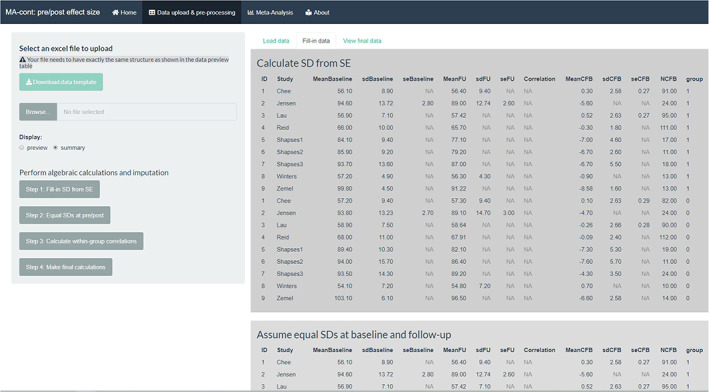
Screenshot of the output of the ‘Fill‐in’ tab, where each *action button* performs a calculation/imputation task [Colour figure can be viewed at wileyonlinelibrary.com]

**FIGURE 5:**
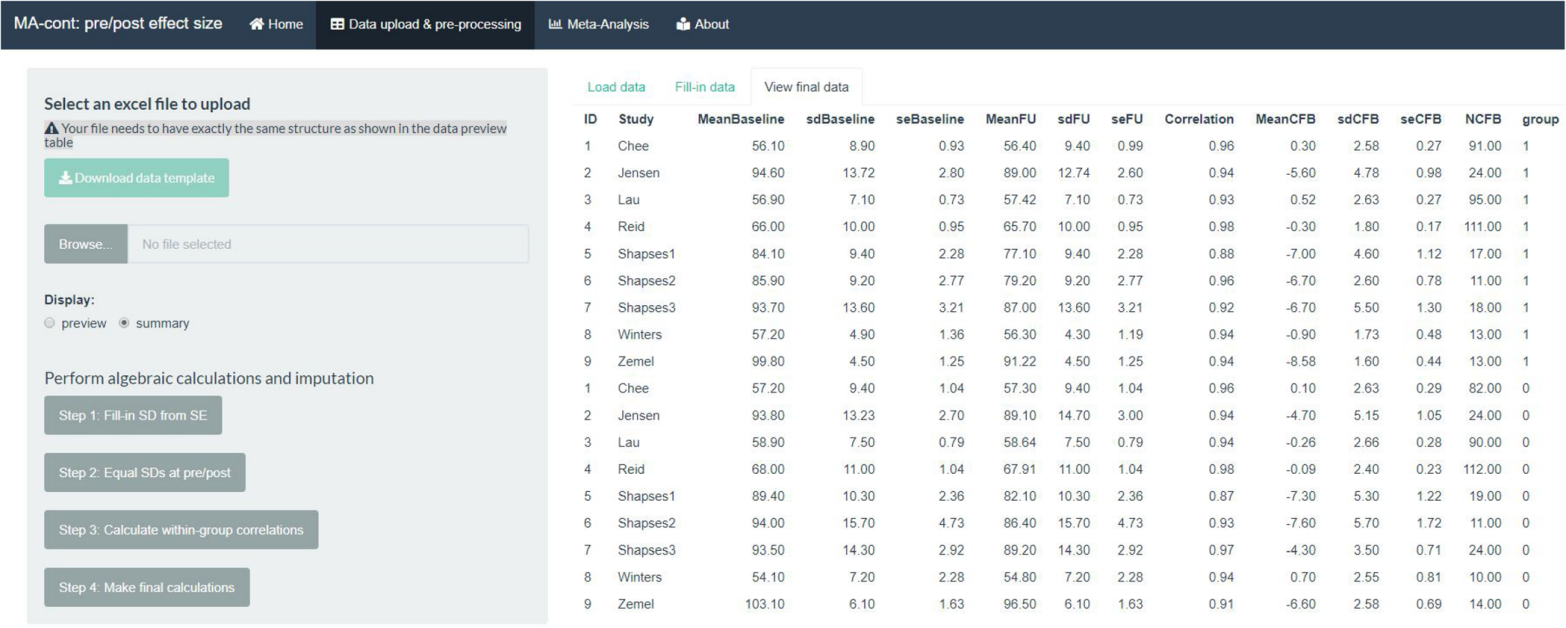
Screenshot of the output of the ‘View final data’ tab, where a complete data set is created [Colour figure can be viewed at wileyonlinelibrary.com]

The sequence of filling‐in steps is shown in Figure 4. The last *action button* performs any final calculations necessary to create a complete data set, which is then used for the analysis. This final data set can be viewed under the ‘View final data’ tab as shown in Figure 5.

Step 3: Choose a meta‐analysis model for an AD analysis.

**FIGURE 6:**
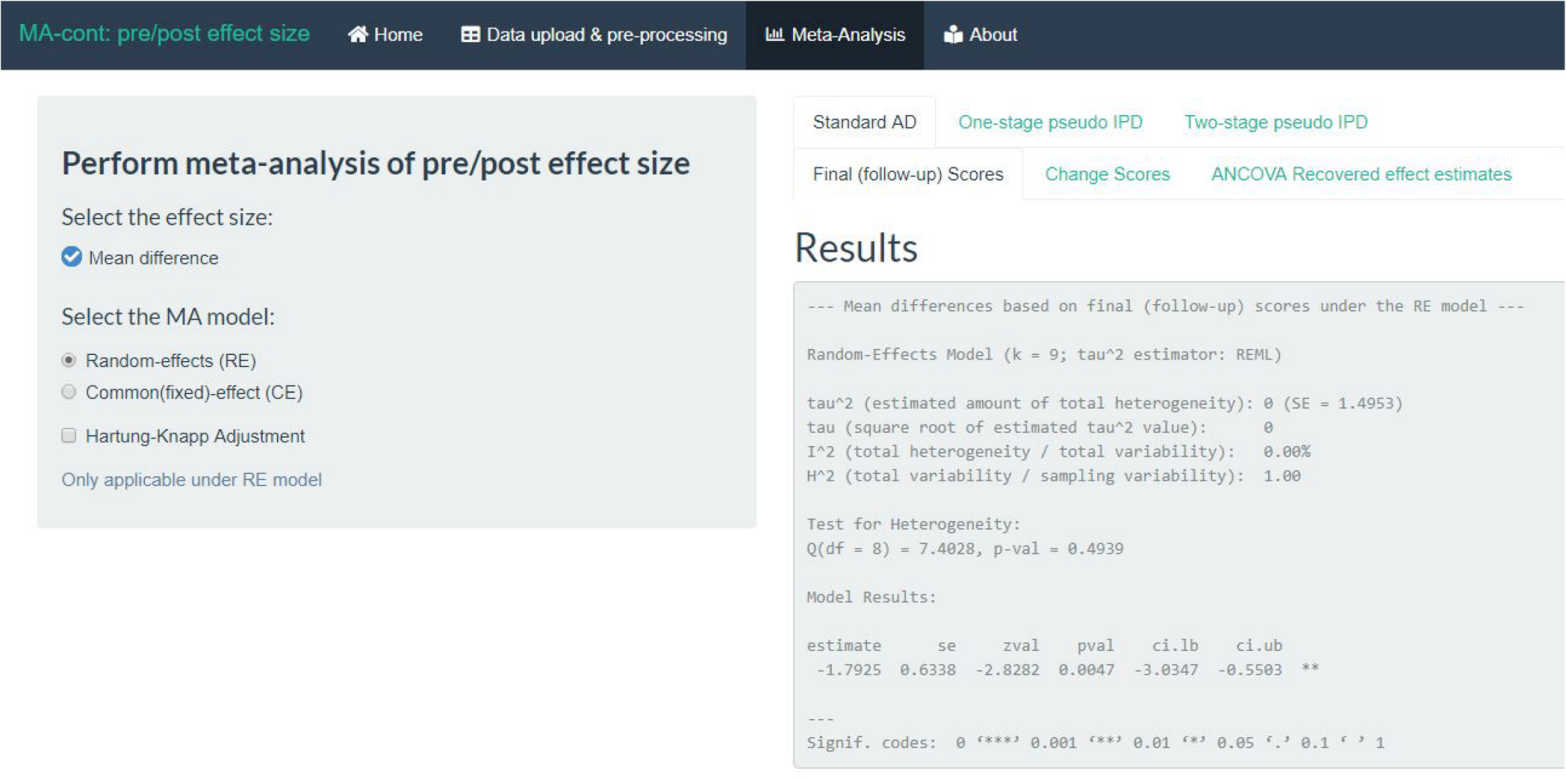
Screenshot of the available meta‐analysis models [Colour figure can be viewed at wileyonlinelibrary.com]

Once a complete data set is created, the next step is to navigate to the ‘Meta‐Analysis’ tab under which a plethora of modelling approaches are offered to the user. This page is structured as such to provide options to select the type of meta‐analysis model and additional functionalities, as shown in Figure 6. The default choice of effect size is set to ‘Mean difference’ since the most appropriate methods for meta‐analysis of pre/post measurements have been, thus far, proposed only for the mean difference.^17^, ^34^ Additional efforts may be put in the future by the developer and contributors of this tool to incorporate other effect size measures, for example standardised mean differences (SMDs).

The tool is organised as such to offer three key analytic approaches under the respective tabs: ‘Standard AD’, ‘One‐stage pseudo IPD’ and ‘Two‐stage pseudo IPD’. The first tab of the standard AD approaches is split into three sub‐tabs, which perform the routinely used final (follow‐up) scores and change scores methods and the ANCOVA recovered estimates method. The first interaction step of the user with the app is to choose between a RE or a CE meta‐analysis model (default option is the RE). It is also possible to implement the recommended HK^25^, ^26^ adjustment method by ticking the respective box. The same selection options apply also to the ‘Two‐stage pseudo IPD’ approach tab.

Step 4: Obtain results.

For the one‐ and two‐stage pseudo IPD approaches the output is split in two sub‐tabs to distinguish between the summary treatment effect and the treatment‐by‐baseline interaction effect results, respectively. For the AD analyses, we provide the results of three analytic methods to estimate the summary treatment effect.

### 4.1 Summary treatment effect

**FIGURE 7:**
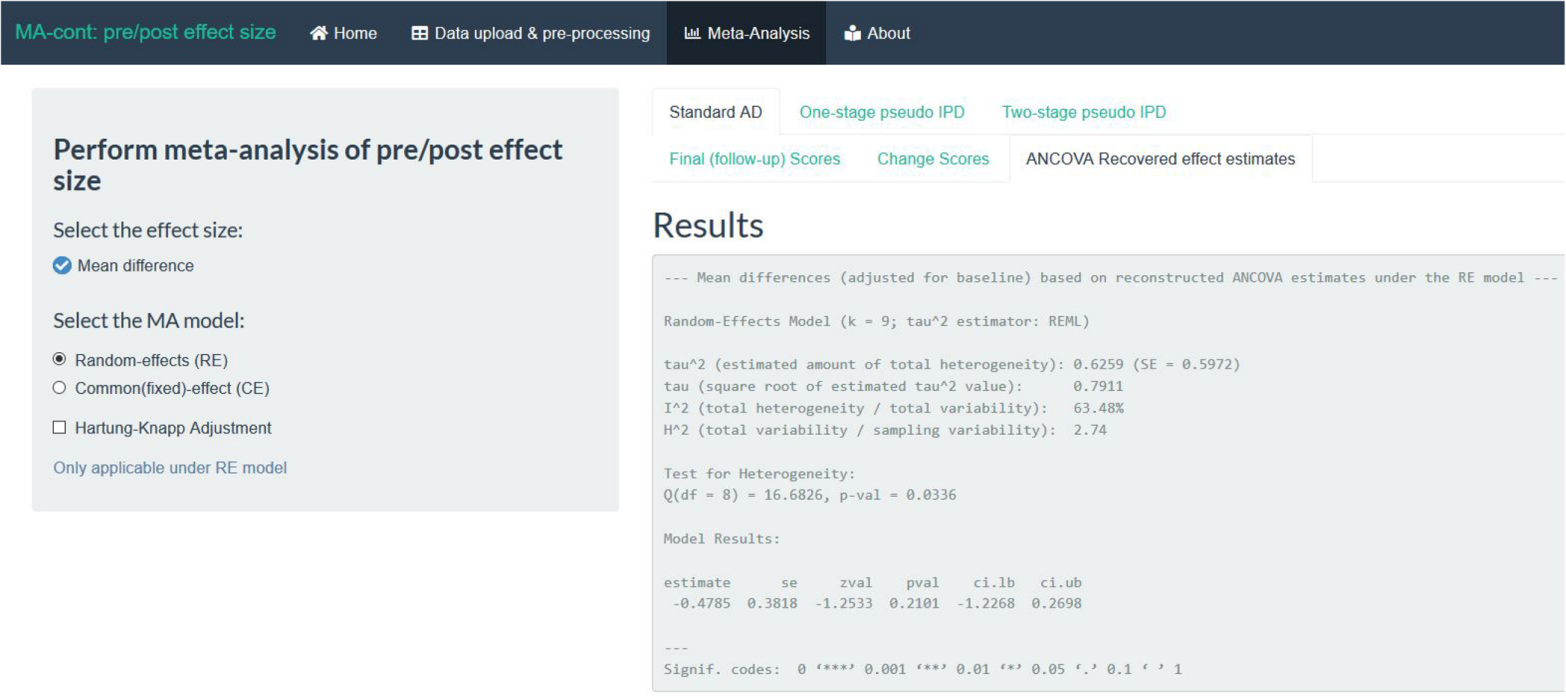
Screenshot of the results based on the ANCOVA recovered estimates approach. [Colour figure can be viewed at wileyonlinelibrary.com]

The generated output under the ‘Standard AD’ tab is identical for the three approaches and consists of a verbose print out and standard visualisations of meta‐analytic results. For example, if we click on the ‘ANCOVA Recovered effect estimates’ (which is more appropriate than final (follow‐up) or change scores analyses), the results are provided in the verbose summary of the *rma* function of metafor. The summary treatment effect is estimated equal to −0.48 kg (95% CI: −1.23, 0.27) (Figure 7). Additional relevant output can be found, for example, the estimate of the between‐study heterogeneity parameter *τ* ^2^, in this case estimated equal to 0.63 and the *I* ^2^ and *H* ^2^ statistics. Identical output and very similar results can be found under sub‐tab ‘Treatment effect’ of the ‘Two‐stage pseudo IPD’ tab. In the worked example, the analysis of final (follow‐up) scores produced a markedly different effect estimate (−1.79 kg) because the method ignores the notable baseline imbalance favouring the active group.

Under the ‘One‐stage pseudo IPD’ tab, we initially provide a data table of the generated pseudo IPD baselines and outcomes of each pseudo participant to enable the users to familiarise themselves with the pseudo IPD approach. Each line is a pseudo IPD observation similar to a true IPD one, and the user can navigate along the various rows of the dataframe. This pseudo IPD set is subsequently used to fit the one‐ and two‐stage ANCOVA methods.

**FIGURE 8:**
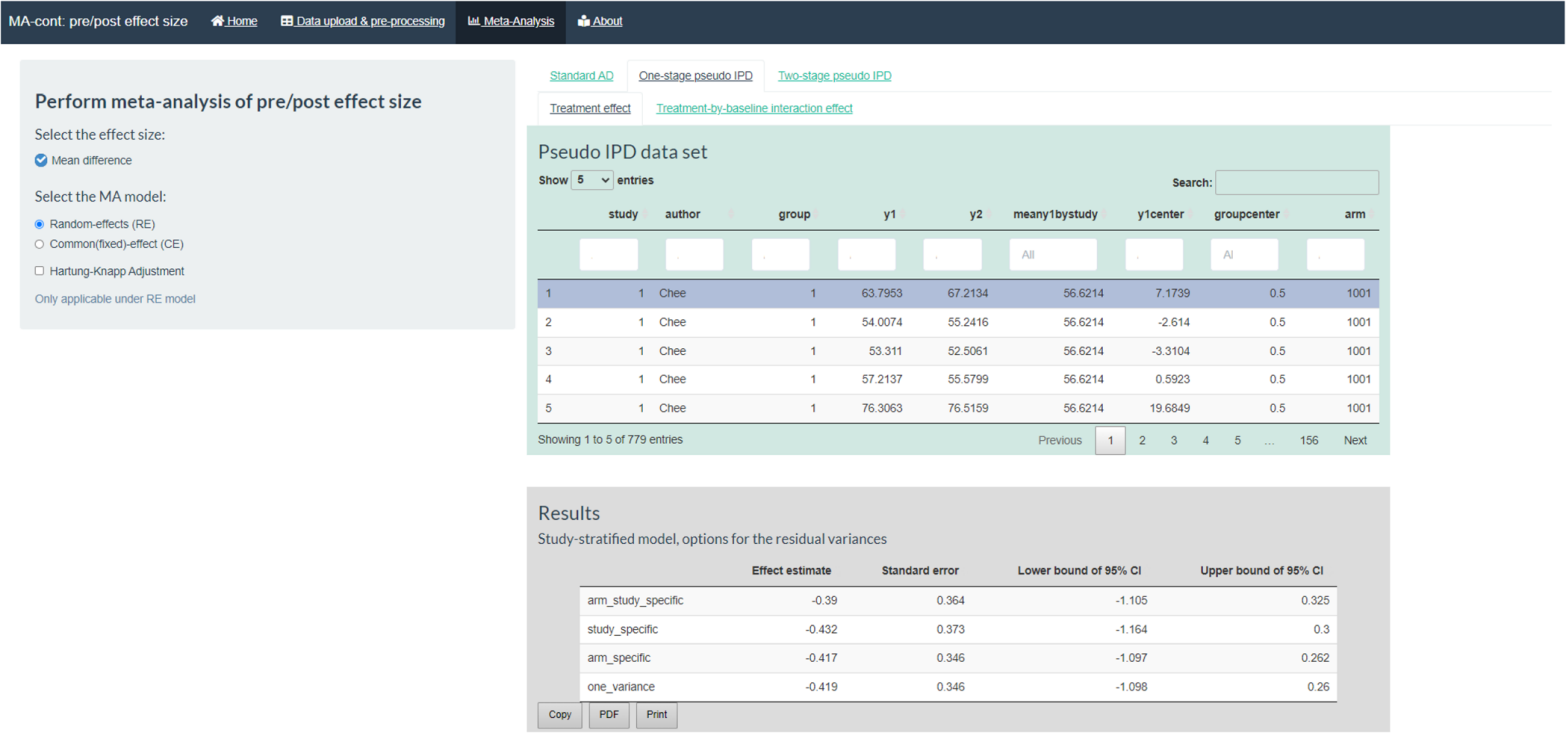
Screenshot of the one‐stage pseudo IPD approach, the pseudo IPD set and the results across the various options for the within‐trial residual variances. IPD, individual participant data [Colour figure can be viewed at wileyonlinelibrary.com]

The results of the summary treatment effect under this approach are given in the second table (in grey) of this tab (Figure 8). We provide four possible options to model the within‐trial residual variances to allow the user to assume more realistic scenarios depending on the data specificities; more details can be found in Table 1. We encourage the user to think which assumption suits his/her data best or present all four, if possible. When interested in a summary estimate that naturally compares with the two‐stage approach or the AD ANCOVA recovered estimates, one should opt for presenting the results from the study‐specific within‐trial residual variance model. In this example, the summary treatment effect was estimated equal to −0.43 kg (95% CI: −1.16, 0.3).

**TABLE 1:** Models for the within‐trial residual variance

Arm‐ and study‐specific $\sigma_{\mathrm{ik}}^{2}$:	Varies by arm and study; the most flexible approach
Study‐specific $\sigma_{i}^{2}$:	Varies by study but is assumed equal between treatment and control arms; to be used when variation of outcomes is expected to be same between the two arms
Arm‐specific $\sigma_{k}^{2}$:	Varies by arm but is assumed equal across all studies; to be used when outcomes are expected to vary between treatment and control arms, particularly when greater variability is expected in the active treatment group
One variance *σ* ^2^:	A single variance parameter as it does not vary across arms or studies

### 4.2 Treatment‐by‐baseline interaction effect

**FIGURE 9:**
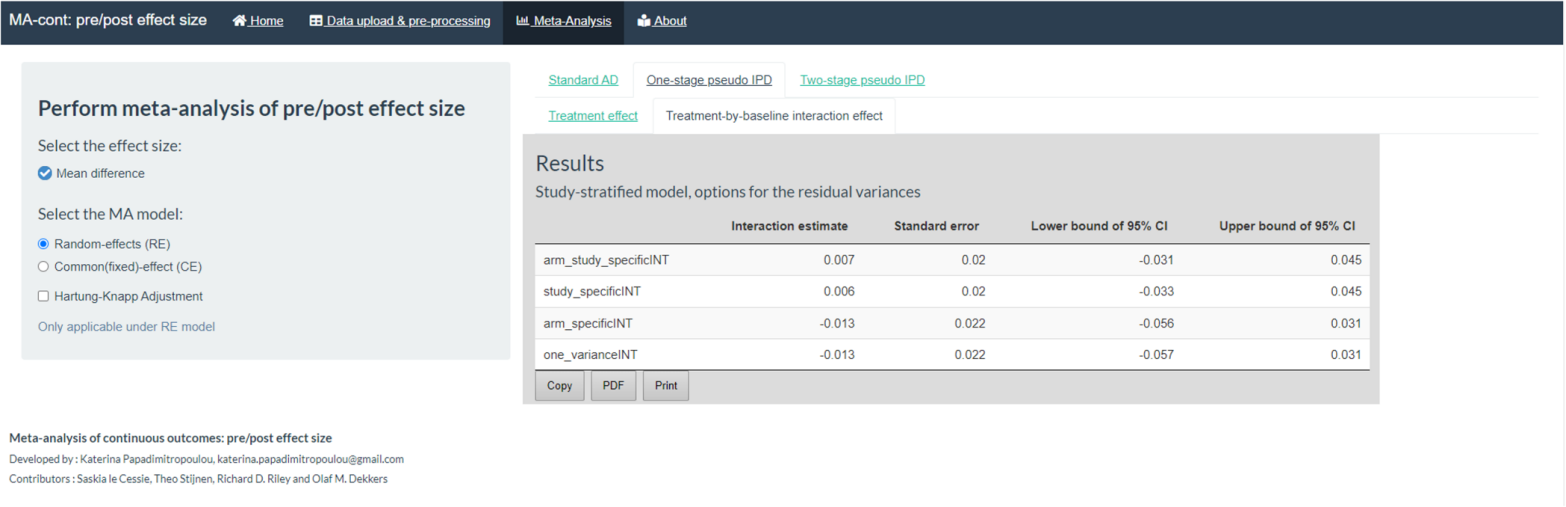
Screenshot of the results for the treatment‐by‐baseline interaction effect under the one‐stage pseudo IPD approach. IPD, individual participant data [Colour figure can be viewed at wileyonlinelibrary.com]

The within‐trial treatment‐by‐baseline interaction effect can be obtained under the homonym sub‐tabs of the ‘One‐stage pseudo IPD’ and ‘Two‐stage pseudo IPD’ tabs. In the first approach, the results are presented in a table, with four distinct options of the within‐trial residual variance (Figure 9). If we focus on the study‐specific assumption for the residual variances, then the interaction effect is estimated equal to 0.006 (95% CI: −0.033, 0.045), indicating a non‐significant relationship at participant level between the baseline score and the treatment effect.

Very similar results may be found under the ‘Two‐stage pseudo IPD’ tab. Under this analysis, it is possible to switch to a common (fixed) interaction effect or to include the HK correction for constructing a 95% confidence interval for the summary interaction effect.

Step 5: Visualise results via forest plots and funnel plots.

**FIGURE 10:**
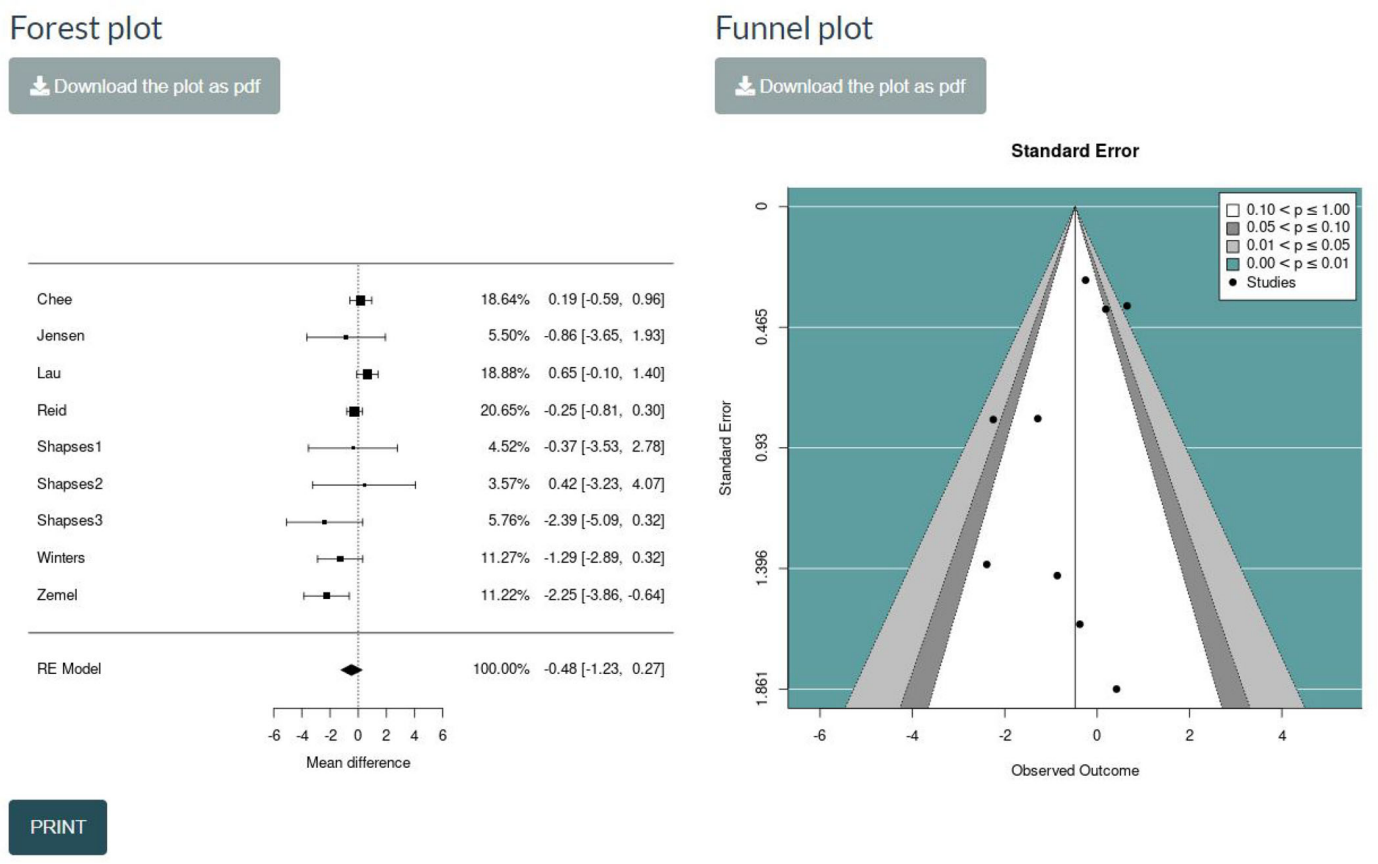
Screenshot of the forest plot and funnel plot for the summary treatment effect RE analysis under the ANCOVA recovered estimates method. ANCOVA, analysis of covariance; RE, random‐effects [Colour figure can be viewed at wileyonlinelibrary.com]

For the standard AD and the two‐stage pseudo IPD approaches, we provide (a) a forest plot, to graphically present the study findings and the summary effect and (b) a funnel plot, to visually inspect potential publication bias. Both visualisations for the ANCOVA recovered effect estimates are shown in Figure 10. Any choices by the user to switch to a CE model or to incorporate the HK approach under the RE model are automatically rendered to the forest and funnel plots. Both plots can be saved in a portable document format (PDF) by clicking at the respective *action buttons*.

Step 6: Save and extract output.

All plots produced by the app can be downloaded as PDF or as a portable network graphic (PNG). In addition, the analyses under the ‘Standard AD’ tab can be saved by clicking on the ‘PRINT’ *action button* at the bottom of the page. This opens a dialogue window where the page (prior being sent to the printer) can be saved in a PDF format. We additionally provide three options to interact with the output of the tables under the ‘One‐stage pseudo IPD’ tab. It is possible to copy the table (and pasting it to a Word document), save it as PDF or send it to the printer, by clicking at the respective buttons.

## 5 DISCUSSION

MA‐cont:pre/post effect size facilitates performing standard AD methods and more sophisticated analyses of continuous outcome data measured at baseline and follow‐up, in a straightforward manner. It is a freely available tool, aspiring to attract a wide audience of technical and non‐technical meta‐analysts. Its development is rooted in the need to tackle the barrier of statistical expertise and software fluency to perform the most appropriate method to meta‐analyse such data, that is the ANCOVA.

A large toolbox of methods is offered to the user and while simple ‘pointing and clicking’ can produce a handful of results, we encourage the user to treat the results with critical thinking. When possible, we suggest to consult a statistician or a meta‐analysis expert to offer help in interpretation of conclusions or modelling assumptions. It is a conscious choice to supply more than one meta‐analysis method to educate and draw attention to the possibility of obtaining conflicting or markedly different results by the available methods (as found when analysing the default data set of Trowman et al.^35^). As discussed throughout this paper, we recommend to undertake ANCOVA approaches to synthesise randomised trials measuring continuous outcomes at baseline and follow‐up time points. Our preference lies with the pseudo IPD ANCOVA approach, implemented in a one‐ or two‐stage fashion for being more flexible and offering exploration of effect modification. The AD ANCOVA recovered estimates approach is a good alternative, when (a) treatment effect modification is not anticipated and (b) equal within‐trial variances may be assumed.

The landscape of free‐to‐use and commercial software to perform meta‐analysis is vast, especially in the medical field. We do not wish to provide an exhaustive overview of existing software options yet some comparisons to MA‐cont:pre/post effect size are more natural. We restrict the space to freely available software and to user‐friendly interfaces, which do not require knowledge of any programming or statistical language, for example R. The meta‐analysis via Shiny (MAVIS)^36^ performs meta‐analysis of continuous outcomes using only follow‐up scores and cannot handle baseline measurements. It is a great tool with graphical functionalities similar to our newly developed app, however it requires manual input of the data (copy/paste from excel file). Similar software (using R in backend but not requiring knowledge of R), are the meta‐analysis function of JASP^37^ and the MAJOR module by jamovi.^38^ Both are rather new and exciting programs, sharing some of the same creators and heavily relying on metafor functionalities. Neither program offers ANCOVA approaches (AD or pseudo IPD options) nor the recommended HK approach to estimate the standard error of the summary effect. In addition, at the moment, the effect sizes need to be entered in JASP, as the software cannot compute them from group‐level summary data. Hence, these software options while offer similar functionalities and a ‘point and click’ interface as the shiny environment, do not offer the core statistical approach which distinguishes MA‐cont:pre/post effect size.

Building shiny apps has grown in popularity and its community of developers and users is getting larger acknowledging how powerful this tool can be in education, research, industry (by deploying large scale tools) and more. In the evidence synthesis setting, we distinguish examples of shiny tools (with different scopes than ours), for example, MetaInsight for network meta‐analysis,^39^ MetaDTA ^40^ and IPDmada ^41^ for meta‐analysis of AD and IPD diagnostic tests, respectively and robvis for risk‐of‐bias assessment.^42^

Our tool may also be improved and extended and we welcome any suggestions by the readers/users. A natural extension would accommodate additional effect sizes, for example, the SMD or the ratio of means. However, this extension requires methodological work prior to software implementation. Recently there is criticism in the meta‐analytic literature concerning the use of SMDs due to the sample‐based standardisation^18^, ^43^, ^44^, ^45^ and thus our recommendation would be to map the outcomes to a common scale and then upload the data in the tool and proceed with the analyses. In addition, the potential of sensitivity analysis by varying the values of the imputed missing statistics, for example, within‐group correlations can be explored in the future.

## AUTHOR CONTRIBUTIONS

KP is the guarantor for the article. All authors provided a substantial contribution to the design of the app, as well as reviewing, editing and approving the final version the manuscript. KP developed the app.

## CONFLICT OF INTEREST

The authors declare no potential conflict of interest.

## Data Availability

The software and data presented in this paper are freely available on Github: https://github.com/Katerina-Pap/MA-cont-shiny-app
